# RASSF1C regulates miR-33a and EMT marker gene expression in lung cancer cells

**DOI:** 10.18632/oncotarget.26498

**Published:** 2019-01-04

**Authors:** Yousef G. Amaar, Mark E. Reeves

**Affiliations:** ^1^ Surgical Oncology Laboratory, Loma Linda VA Medical Center, Loma Linda, CA, USA; ^2^ Loma Linda University Cancer Center, Loma Linda, CA, USA

**Keywords:** lung cancer, RASSF1C, miR-33a, EMT

## Abstract

RASSF1C functions as an oncogene in lung cancer cells by stimulating proliferation and migration, and reducing apoptosis. Further, RASSF1C up-regulates important protein-coding and non-coding genes involved in lung cancer cell growth, including the stem cell self-renewal gene, *piwil1*, and small noncoding PIWI-interacting RNAs (piRNAs). In this article, we report the identification of microRNAs (miRNAs) that are modulated in lung cancer cells over-expressing RASSF1C. A lung cancer-specific miRNA PCR array screen was performed to identify RASSF1C target miRNA-coding genes using RNA isolated from the lung cancer cell line H1299 stably over-expressing RASSF1C and corresponding control. Several modulated miRNA genes were identified that are important in cancer cell proliferation and survival. Among the miRNAs down-regulated by RASSF1C is miRNA-33a-5p (miRNA-33a), which functions as a tumor suppressor in lung cancer cells. We validated that over-expression of RASSF1C down-regulates miR-33a expression and RASSF1C knockdown up-regulates miR-33a expression. We found that RASSF1C over-expression also increases β-catenin, vimentin, and snail protein levels in cells over-expressing miR-33a. In addition, we found that RASSF1C up-regulates the expression of ABCA1 mRNA which is a known target of miR-33a. Our findings suggest that RASSF1C may promote lung epithelial mesenchymal transition (EMT), resulting in the development of a lung cancer stem cell phenotype, progression, and metastasis, in part, through modulation of miR-33a expression. Our findings reveal a new mechanistic insight into how RASSF1C functions as an oncogene.

## INTRODUCTION

Lung cancer is the most common and lethal of all cancers in the United States [1]. Our laboratory has focused on the pro-oncogenic activities of the Ras Association Domain Family Member 1 (*RASSF1)* gene. RASSF1 encodes two major isoforms, RASSF1A and RASSF1C, derived by alternative promoter selection and mRNA splicing [2, 3]. While RASSF1A is a well characterized tumor suppressor [2–6], the RASSF1C isoform appears to function as an oncogene promoting lung cancer cell proliferation, migration, and survival [7–12]. In previously published work we showed that RASSF1C regulates the expression of PIWIL1, a stem cell renewal gene, and small non-coding PIWI-interacting RNAs (piRNAs). This suggests that RASSF1C may promote lung cancer stem cell (CSC) development and progression, in part, through a novel PIWIL-piRNA pathway [7, 12, 19]. We have identified several novel RASSF1C target piRNA genes with either potential tumor-promoting or tumor-suppressing functions [12]. We also showed that RASSF1C may modulate PIWIL1-piRNA gene expression, in part, through promoting ERK1/2 phosphorylation and attenuating the AMPK pathway and downstream effectors p21 and p27 [8, 10, 12].

To further characterize the role of RASSF1C in lung cancer cell growth, we performed a lung cancer-specific microRNA (miRNA) PCR array screen to identify RASSF1C target miRNA-coding genes in non-small cell lung cancer (NSCLC) cells. MiRNAs function as critical post-transcriptional regulators of gene expression. In this article, we report on the identification of several RASSF1C miRNA target genes in NSCLC cells. Among the miRNAs down-regulated by RASSF1C is miR-33a, which is an inhibitor of lung epithelial mesenchymal transition (EMT) and a known lung cancer tumor suppressor [13–15]. MiR-33a down-regulates the expression of pro-EMT genes such as β-catenin, ATP-binding cassette transporter 1 (ABCA1), and vimentin [13]. Here, we report that RASSF1C attenuates miR-33a expression and promotes EMT marker gene expression in NSCLC cells.

## RESULTS

### RASSF1C down-regulates miR-33a gene expression

**Figure 1:**
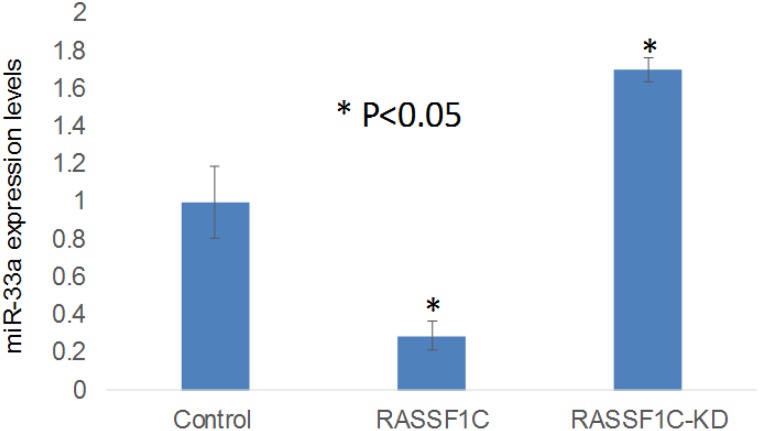
RT-PCR validation of miR-33a-5p expression in the lung cancer cell line H1299 treated with scrambled miRNA (Control), H1299 cells over-expressing RASSF1C (RASSF1), and H1299 cells with RASSF1C-knockdown (RASSF1C-KD) RASSF1C over-expression down-regulates, and silencing up-regulates, the expression of miR-33a-5p in NSCLC cells. The RT-PCR of controls and experimental reactions were run in triplicate in multiple runs and the 2^-ΔΔCT^ method was used to perform statistical analysis [16].

In previous work we have shown that RASSF1C appears to function as an oncogene in lung cancer cells, in part, through a novel RASSF1C-PIWIL1-piRNA pathway which may promote cancer stem cell growth and progression. To further our understanding of how RASSF1C acts as an oncogene, we assessed the impact of RASSF1C on miRNA gene expression. To accomplish this, we performed a miRNA RT-PCR array screen using total RNA isolate from the lung cancer cell line NCI-H1299 over expressing RASSF1C and NCI-H1299 over-expressing vector backbone to identify miRNAs that are regulated by RASSF1C. The lung cancer miRNA PCR array screen has identified several interesting miRNA target genes that are modulated by RASSF1C. The miRNA genes that are up- or down-regulated by RASSF1C include some that impact tumor growth (Table 1). Among the miRNAs down-regulated by RASSF1C is miR-33a, which is known to inhibit EMT and to suppress cancer cell growth [13, 14]. We are interested in RASSF1C regulation of miR-33a expression because RASSF1C could promote EMT through a mechanism that involves up-regulation of PIWIL1-piRNA gene expression and down-regulation of miR-33a expression. We, therefore validated that over-expression of RASSF1C leads to significant down-regulation, and silencing of RASSF1C leads to significant up-regulation of miR-33a expression in lung cancer cells (Figure 1). This novel finding that RASSF1C is a negative regulator of miR-33a gene expression suggests a potential role for RASSF1C in promoting lung EMT.

**Table 1:** Selected RASSF1C miRNA target genes identified using lung cancer specific miRNA PCR array

Up-regulated miRNAs Fold change > 5	Down-regulated miRNAs Fold change > -5
hsa-miR-15a-5p	hsa-miR-33a-5p
hsa-miR-21-5p	hsa-miR-98
hsa-mIR-31-5p	hsa-miR-107
Has-miR-125	hsa-miR-128

### RASSF1C attenuates miR-33a effects on pro-EMT genes

**Figure 2:**
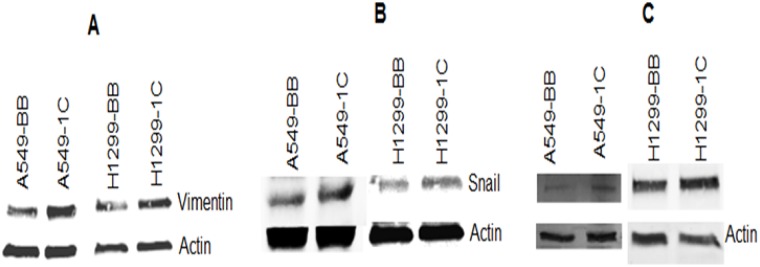
RASSF1C up-regulates the expression of vimentin, β-catenin, and snail in the NSCLC cell lines A549 and H1299 Vimentin (**A**), β-catenin (**B**), and snail (**C**) protein levels are higher in A549-1C and H1299-1C cells over expressing RASSF1C compared to A549-BB and H1299-BB cells transfected with vector backbone.

**Figure 3:**
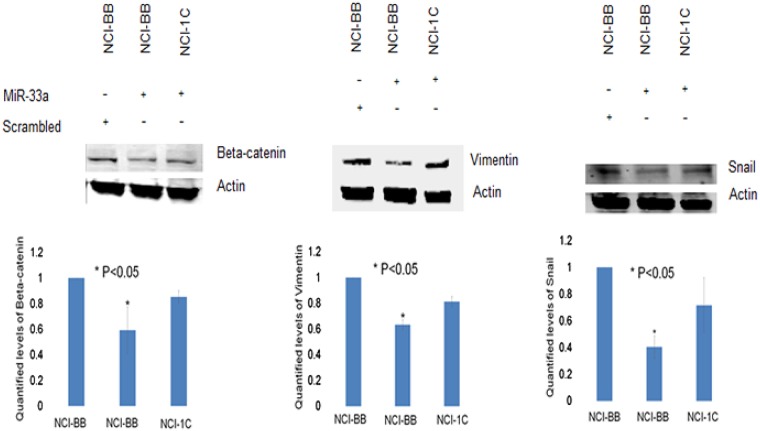
RASSF1C attenuates the effects miR-33a-5p on EMT marker genes Treatment of H1299-vector backbone (H1299-BB) or H1299-overexpressing RASSF1C (NCI-1C) with miR-33a-5p mimics reduced protein levels of β-catenin, vimentin, and snail in control cells (NCI-BB) but to a lesser extent in cells over-expressing RASSF1C when compared to NCI-BB cells transfected with scrambled miRNA. Quantified levels of vimentin, β-catenin, and snail were determined as an average signal (NCI-BB vs NCI-1C) from at least 3 independent blots, with a *P* < 0.05.

**Figure 4:**
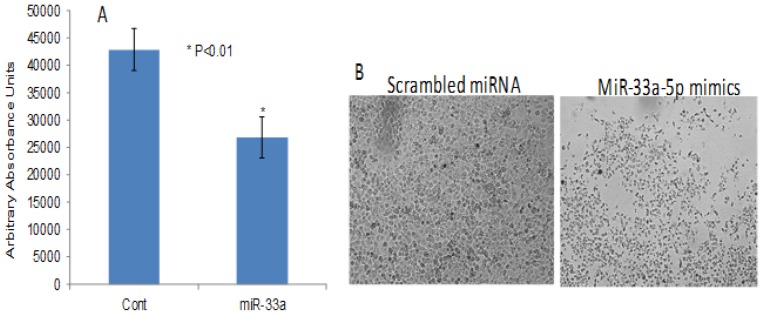
MiR-33a-5p over-expression reduces lung cancer cell proliferation and colony formation The NSCLC cell line H1299 was treated with 100 nM of miR-33a5p mimics or scrambled miRNA oligos for 48 h, and the miR-33a-5p mimics reduced cell proliferation (**A**). All experiments were done at least 3 independent times with *n* = 4 wells per treatment. The (^*^) indicates statistical significance compared to controls (scrambled miRNA), with a *P* < 0.05. (**B**) Shows the impact of miR-33a-5p mimics on H1299 colony formation after 72 h incubation post transfection.

**Figure 5:**
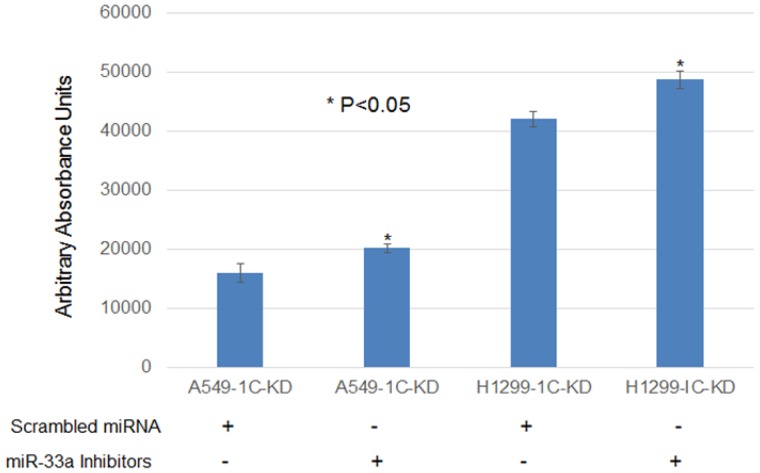
MiR-33a-5p down-regulation resulted in a statistically significant increase (15–20%) in cell proliferation of lung cancer cells A549 and H1299 with RASSF1C knocked down The A549 and H1299 cell lines with RASSF1C Knocked down were treated with 100 nM of scrambled miRNA oligos or miR-33a-5p inhibitors for 48 h, Cell viability/proliferation was measured using the Alamar Blue assay. Cell transfection were down an *n* = 4 wells at least 3 independent times. The (^*^) indicates statistical significance compared to controls (scrambled miRNA), with a *P* < 0.05.

To further investigate the impact of RASSF1C on the down-regulation of miR-33a expression, we assessed the expression of EMT marker genes that are known to be down-regulated by miR-33a. it is known that miR-33a down-regulates the expression of β-catenin, vimentin, and snail [13]. MiR-33a bind to the 3UTR region of β-catenin and leads to down-regulation of β-catenin expression and prevents it translocation into the nucleus [17–21]. It is also known that RASSF1C promotes β-catenin expression in normal lung epithelial and lung cancer cell lines [10, 23]. Thus, we wanted to determine if RASSF1C over-expression will attenuate the effects of miR-33a on β-catenin as well as vimentin and snail in lung cancer cells. Here, we first found that RASSF1C promotes the expression of β-catenin, vimentin, and snail in lung cancer cells (Figure 2). Second, we assessed the impact of miR-33a on the expression of β-catenin, vimentin, and snail in cells over-expressing RASSF1C. We found that over-expression of miR-33a in lung cancer cells down-regulates the expression of β-catenin, vimentin and snail (Figure 3). However, RASSF1C over-expressing cells treated with miR-33a show higher expression levels of β-catenin, vimentin, and snail compared to control cells treated with miR-33a mimics (Figure 3). We also treated, lung cancer cells with miR-33a mimics and scrambled control to verify that transfection of lung cancer cells with miR-33a mimics decreases cell proliferation and colony formation (Figure 4), which is consistent with published literature [13, 14]. Further, we found that miR-33a inhibitors resulted in a small but statistically significant increase in cell proliferation of lung cancer cell lines with RASSF1C knocked down (Figure 5). Our findings suggest that RASSF1C may promote lung cell EMT, in part, through down-regulation of miR-33a expression which in turn up-regulates β-catenin and EMT marker genes such as vimentin and snail.

### RASSF1C up-regulates ABCA1 gene expression which is down-regulated by miR-33a

**Figure 6:**
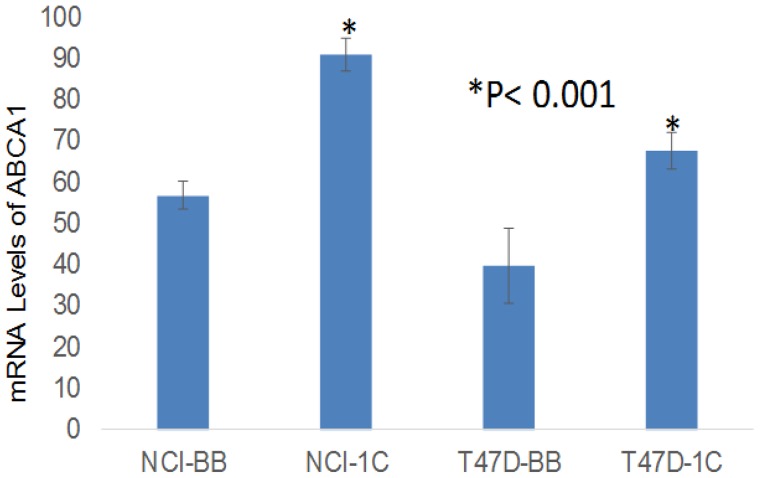
Microarray data show that over-expression of RASSF1C in the lung cancer cell line H1299 (NCI-1C) and in the breast cancer cell line T47D (T47D-1C) up-regulates ABCA1 gene expression compared to controls (NCI-BB and T47D-BB) The (^*^) indicates statistical significance compared to controls (scrambled miRNA), with a *P* < 0.01.

**Figure 7:**
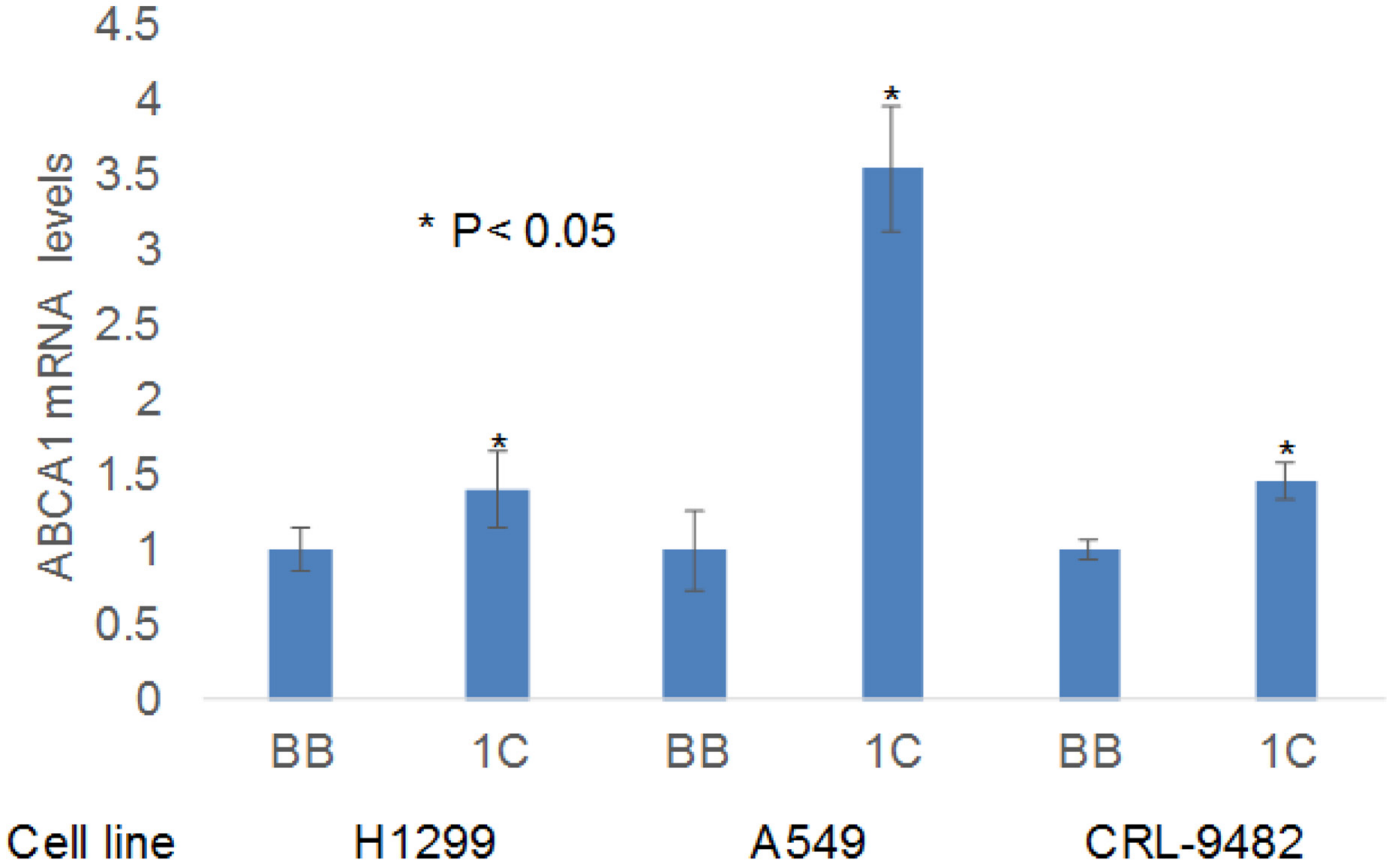
RT-PCR validation of ABCA1 expression RASSF1C over-expression up-regulates ABCA1 gene expression in the NSCLC cell lines H1299 and A549 and in the normal lung epithelial cell line CRL-9482. The RT-PCR was performed in triplicate in multiple runs and the 2^-ΔΔCT^ method was used to perform statistical analysis [16], with a *P* < 0.05.

It has also been reported that miR-33a binds the 3’UTR-region of ATP drug transporter gene ABCA1 leading to cleavage of the ABCA1 mRNA. In previously published work, we showed that RASSF1C promotes drug resistance of both lung and breast cancer cells [10, 11]. Thus, we wanted to know if RASSF1C over-expression also up-regulates ABCA1 which has been linked to cancer cell drug resistance [20, 21]. Using data from our previous Microarray studies [8, 11], we checked the expression levels of ABCA1 in cell lines over-expressing RASSF1C and we found that RASSF1C over-expression in both breast and lung cancer cell lines resulted in a significant increase in the expression level of ABCA1 (Figure 6). The up-regulation of ABCA1 was validated in normal lung epithelial and lung cancer cells by RT-PCR (Figure 7). Our findings suggest that RASSF1C may promote cancer cell drug resistance through up-regulation of the ABCA1 gene expression and down-regulation of miR-33a expression.

## DISCUSSION

In previously published work, we demonstrated that RASSF1C acts as an oncogene through the modulation of several protein-coding and small non-coding PIWI-interacting RNAs (piRNAs) that are associated with promoting cell proliferation and migration, and attenuating apoptosis [7–12]. In this study we show that RASSF1C also modulates the expression of important miRNAs (Table 1). Among the miRNAs down-regulated by RASSF1C is miR-33a. This is very important because of the known relationship between miR-33a and cancer. For instance, miR-33a is known to suppress lung cancer cells growth *in vitro* [15, 24]. Further, miR-33a expression is down-regulated in lung tumor tissues compared to matched normal tissue, and miR-33a expression is positively correlated with overall patient survival [25]. In the present study, we found that over-expression of RASSF1C resulted in a significant decrease in miR-33a expression in lung cancer cells, and silencing of RASSF1C resulted in a significant increase in miR-33a. Interestingly, the microarray data showed that RASSF1C over-expression in lung cancer cells did not affect the expression of the miR-33a host gene [22], SREBP2 (data not shown). This suggests that RASSF1C may regulate miR-33a post-transcriptionally. Thus, our findings provide a new mechanism for regulating miR-33a in lung cancer cells by RASSF1C.

Mechanistically, miR-33a suppresses lung cancer cell growth and migration, in part, by inhibiting lung EMT [13, 14]. Consistent with this, we found that treatment of lung cancer cells with miR-33a mimics decreased cell proliferation and expression of β-catenin, vimentin, and snail in lung cancer cells. In addition, miR-33a over-expression has been shown to reduce, and miR-33a silencing to enhance, NSCLC cell migration suggesting that miR-33a may attenuate metastasis of NSCLC cells [13]. In contrast, RASSF1C over-expression has been demonstrated by us and others to up-regulate β-catenin gene expression in lung cancer cells [10, 23]; and in this study, we also show that lung cancer cells over-expressing RASSF1C exhibit increased vimentin and snail protein levels. Indeed, the impact of miR-33a over-expression on β-catenin, vimentin, and snail protein levels is less pronounced in lung cancer cells over-expressing RASSF1C (Figure 3) as one would predict. We also found that inhibiting miR-33a expression in lung cancer cells with RASSF1C knocked down enhances cell proliferation (Figure 5). Also pertinent is that we have previously shown that RASSF1C promotes cell migration of NSCLC and breast cancer cells [10, 11]. Taken together our current and previous findings suggest that RASSF1C may promote lung EMT, cell proliferation, and cell migration, in part, through down-regulation of miR-33a.

We also found that RASSF1C up-regulates the ABCA1 gene which is another gene that is known to be a target for miR33a. The 3’-UTR of ABCA1 mRNA contains a miR-33a binding site, and thus miR-33a suppresses ABCA1 expression. Consistent with this, we show here that RASSF1C down-regulates miR-33a while it up-regulates ABCA1 expression (Figures 6, 7). Interestingly, we have shown in previously published work that RASSF1C over-expression desensitizes breast and lung cancer cells to the apoptotic effects of Etoposide [10] and betulinic acid [11], respectively. Indeed, ABCA1 has been reported to mediate drug resistance in cancer cells [21, 26, 27]. Thus, it is possible that RASSF1C down regulation of miR-33a results in up-regulation of ABCA1 gene expression could be a mechanism through which RASSF1C enhances breast and lung cancer cell drug resistance.

**Figure 8:**
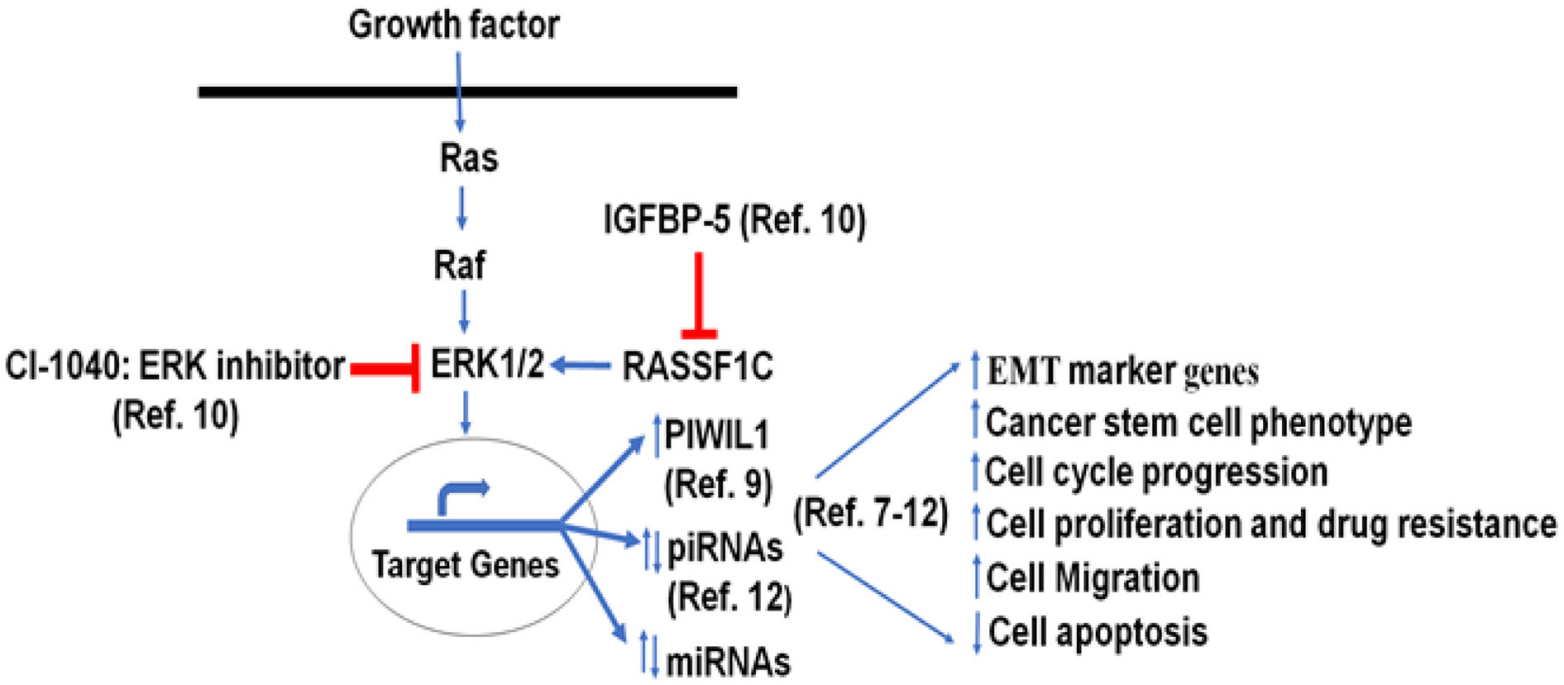
Hypothetical model: previous and current miRNA work suggest that RASSF1C modulation of the PIWIL1-piRNA and miRNA gene axis in lung cells may contribute to lung cancer stem cell development and progression [7–12].

Our previous work suggests that RASSF1C may promote lung cancer stem cell development and progression, in part, through modulation of a novel PIWIL1-piRNA pathway [10]. PIWIL1 promotes endometrial cancer stem cell development through endometrial EMT as over-expression of PIWIL1 increases N-cadherin and vimentin and decreases E-cadherin gene expression [26]. Also, EMT has been associated with promoting cancer stem cells in solid tumors [27–31]. We believe that RASSF1C up-regulation of EMT marker genes and ABCA1 gene expression, in part through down-regulation of miR-33a, provides novel mechanistic possibilities of how RASSF1C may contribute to lung EMT/cancer stem cell development and progression through modulation of a PIWIL1-piRNA and miRNA gene axis. Figure 8 depicts a hypothetical work model based on our previous and current findings. Future planned mechanistic studies will test this model both *in vitro* and *in vivo*.

## MATERIALS AND METHODS

### Cell culture

The human lung cancer cell lines NCI-H1299 and A549 and normal lung epithelial cell line CRL-9482 were obtained from American Type Culture Collection (Manassas, VA). NCI-H1299, A549, and CRL9492 cells stably transduced with MLV-backbone (BB) and MLV-HA-RASSF1C (1C) were used as previously described [10]. H1299 cells over-expressing sh-RNA specific to RASSF1C and H1299 over-expressing scrambled sh-RNA were used as previously described [10]. NCI-H1299 cells were grown in RPMI-1640 medium supplemented with 10% calf bovine serum, A549 cells were grown in F-K12 media supplemented with 10% FBS, and CRL9482 cells were grown in BEMB media [10].

### Total and microRNA isolation

Total RNA and miRNA were isolated from NCI-H1299, A549, and CRL-9482 cells stably over-expressing RASSF1C, H1299 cells stably expressing shRNA specific to RASSF1C, and H1299 cells transfected with the control vector back bone using a total and miRNA isolation kits (Qiagen, Valencia, CA, USA).

### MicroRNA PCR array screen

Lung cancer miProfile™ miRNA PCR Array (Cat. No. QM011, GeneCopoeia, Rockville, MD, USA) was used to identify RASSF1C miRNA target genes. The miRNA cDNA was prepared from mature miRNA isolated from H1299 cells over-expressing RASSF1C and from control cells transfected with the vector backbone. The miRNA PCR array screen was performed according to the user manual. Data analysis was performed with GeneCopoeia's online data analysis software.

### Microarray analysis

Microarray analysis was conducted as previously described [8]. Levels of mRNA expression in control cells expressing the vector backbone (H1299-BB and T47D-BB) and in experimental cells stably over-expressing RASSF1C (H1299-1C and T47D-1C, performed in triplicates, were compared [8].

### Silencing of RASSF1C expression in lung cancer cells

RASSF1C was silenced using Mission Non-Target shRNA Control Transduction Particles or with multiple Mission Lentiviral sh-RNA Transduction Particles (NMID: NM_007182, Sigma, St. Louis, MO, USA) as previously described [8].

### RT-PCR analysis

Total RNA from experimental and control cell lines was isolated from cultures and reverse transcriptase (RT)-PCR was performed using RASSF1C gene-specific primers as previously described [10]. PCR was carried out using the Sybrgreen master mix (Qiagen, Valencia, CA, USA), and the PCR reactions were run with the following conditions: 95°C for 15 min, 95°C for 1 min, 60°C for 30s, and 72°C for 30s for 35 cycles. Amplification of cyclophilin using gene specific PCR primers was used as a loading control. For miRNA expression analysis, reverse transcription was performed using Quantimir RT kit (System Biosciences, Mountain View, CA, USA). PCR was performed using KAPA SYBR^R^ FAST qPCR Kit (KAPA Biosystems, Boston, MA, USA). The first 21 nucleotides of the miRNA sequence were used as a forward primer, along with a universal reverse primer included in the Quantimir RT kit. miR-33A-5p: 5’-GTGCATTGTAGTTGCATTGCA-3 forward primer was used. The RT-PCR reactions were carried out in triplicates and the fold change was calculated using the 2^-ΔΔCT^ method [16]. The RT-PCR runs were repeated at least 3 times.

### Cell transfection

Synthetic miR-33a mimics, miR-33a inhibitors, and scrambled miRNA oligos were obtained from Sigma (Sigma, St. Louis, MO, USA) Transfection of cells with miR-33a mimic, miR-33a inhibitors, and scrambled oligos at 100 nM final concentration was carried out using lipofectamine (Invitrogen, Carlsbad, CA, USA). Cells were plated at 2500–5000 cells per well (*n* = 8), transfected the next day, and were incubated for 48 h before performing the Alamar blue assay. For cell lysate preparations, Cells were plated at 50,000 per well (*n* = 3) in 6-well plates and cells were transfected the next day with 100 nM miR-33a mimics or scrambled miRNA oligos. They were then incubated for 48 h before preparing cell lysates for Western blot analysis using RIPA buffer supplemented with protease inhibitors.

### Cell proliferation assay

Cell proliferation/viability was measured by the Alamar Blue assay. Cells transfected with miRNA scrambled, miRNA mimics, and inhibitors were assayed for cell proliferation using the Alamar blue assay as previously described [7]. Data are presented as mean values ± SEM and analyzed with Student's *t*-test. Values ≤ 0.05 were considered significant. The experiments were repeated at least 3 times.

### Colony formation assay

H1299 cell line were treated with scrambled miRNA oligos (control) or miR-33a mimics or aat a concentration of 100 nM. Cells were plated at 5000 cells per well (*n* = 4) and cells were transfected the next day and incubated for 3 days. Cells were imaged 3 days post-transfection under phase contrast using a wide field imaging system (Leica microsystems Inc, Buffalo Grove, IL, USA).

### Western blot analysis

Western blot analysis of experimental and control cell lysates was carried out using the Odyssey^®^ Infrared System (LI-COR Biosciences, Lincoln, NE, USA). Cell lysate from control and experimental cells were prepared using RIPA lysis buffer supplemented with 1× protease inhibitors (Sigma) and 25 μg of cell lysates were used to run Western blots. β-catenin, vimentin, and snail polyclonal antibodies were purchased from Cell Signaling Technology, Inc. Polyclonal beta actin antibody (Cat # sc-1615) was purchased from Santa Cruz Biotechnology, Inc (Santa Cruz, CA, USA), and fluorescently-labeled secondary antibodies IRDye^®^ 680 and 780 RD Infrared Dye were purchased from LI-COR (LI-COR Biosciences, Lincoln, NE, USA). The experiments were repeated at least 3 times. Protein levels were normalized to actin levels (the loading control).

### Statistical analysis

The *t*-test was used to calculate the significance of data.

## CONCLUSIONS

RASSF1C down-regulates the expression of miR-33a and enhances the expression of key EMT marker genes and the ABCA1 gene. Linking RASSF1C to the regulation of miR-33a gene expression and potentially to the induction of lung EMT are novel findings that contribute to a better understanding of lung cancer stem cell biology.
